# Clinical Outcomes of 111 Patients with Early Onset Idiopathic Scoliosis (EOIS) Receiving Brace Treatment: A Longitudinal Retrospective Cohort Study

**DOI:** 10.3390/jcm13030767

**Published:** 2024-01-29

**Authors:** Rufina Wing-Lum Lau, Alec Lik-Hang Hung, Ho-Man Kee, Leo Chung-Hei Wong, Victor Kin-Wai Chan, Derek Wai-Yin Chung, Jerry Kwok-To Chan, Bosco Kin-Pok Chau, Stanley Ho-Fung Leung, Jack Chun-Yiu Cheng, Tsz-Ping Lam, Adam Yiu-Chung Lau

**Affiliations:** 1 School of Medical and Health Sciences, Tung Wah College, Hong Kong SAR, China; rufinalau@twc.edu.hk; 2 SH Ho Scoliosis Research Lab, Joint Scoliosis Research Center of the Chinese University of Hong Kong and Nanjing University, Department of Orthopaedics & Traumatology, The Chinese University of Hong Kong, Hong Kong SAR, Chinajackcheng@cuhk.edu.hk (J.C.-Y.C.); tplam@cuhk.edu.hk (T.-P.L.); 3 Department of Prosthetics and Orthotic, Prince of Wales Hospital, Hospital Authority, Hong Kong SAR, China; 4 Department of Orthopaedics and Traumatology, The Chinese University of Hong Kong, Hong Kong SAR, China; 1155077497@link.cuhk.edu.hk (L.C.-H.W.); 1155094935@link.cuhk.edu.hk (D.W.-Y.C.); 1155157982@link.cuhk.edu.hk (J.K.-T.C.); 1155159768@link.cuhk.edu.hk (B.K.-P.C.);

**Keywords:** early onset, idiopathic scoliosis, curve progression, in-brace correction, bracing, conservative treatment

## Abstract

**Introduction**: Bracing is one of the first-line treatment for early-onset idiopathic scoliosis (EOIS) to control curves from progression. This study aimed to explore the determinants that govern bracing effectiveness in EOIS. **Methods**: One hundred and eleven patients with EOIS (mean age of 8.6 ± 1.25 at diagnosis) received bracing treatment and had a final follow-up beyond skeletal maturity were identified from records between 1988 and 2021. Demographic data and clinical features of spinal curvature were obtained for correlation analyses to determine the associations between curve outcomes and clinical features. **Results**: Most patients were female (85.6%) and had a major curve on the left side (67%). The mean baseline Cobb angle of major curves was 21.73 ± 7.92°, with a mean Cobb angle progression of 18.05 ± 19.11°. The average bracing duration was 5.3 ± 1.9 years. Only 26 (23.4%) of them underwent surgery. The final Cobb angle and curve progression at the final follow-up with a Cobb angle of ≥50° were positively correlated with the initial Cobb angle (r = 0.206 and r = 0.313, respectively) and negatively correlated with maturity parameters. The lumbar curve type was found to correlate with a smaller final Cobb angle. **Conclusions**: The majority of patients had a final Cobb angle < 50°, which was considered a successful bracing outcome. The final Cobb angle correlated with the initial Cobb angle and curve types observed in EOIS.

## 1. Introduction

Owing to the unclear etiopathogenesis, bracing, scoliosis-specific physiotherapy exercises and instrumental spinal surgery are the only available evidence-based treatment strategies for the management of idiopathic scoliosis. These strategies are employed primarily to control the further deterioration of the resultant spinal deformity rather than the cause. Untreated idiopathic scoliosis, especially early-onset idiopathic scoliosis (EOIS), is more prone to progressive deformities, cosmetic defects, thoracic insufficiency syndrome and increased mortality [1,2].

Early onset scoliosis is defined as >10 degrees of coronal curvature with the onset before 10 years old [3,4]. Although early intervention is crucial to prevent severe or life-threatening sequelae, clinical uncertainty remains among treatment options for EOIS due to the heterogeneous characteristics of this population and the lack of evidence for optimal treatment options [4,5]. Besides life saving and the control of spinal deformity, treatment goals for patients with EOIS also include allowing the growth of the spine, chest wall, and lung development to improve lung function.

Surgical intervention is indicated for severe and progressive curves. Surgical techniques have advanced significantly over the past decade with the use of growth-friendly implants for young children with scoliosis such as the vertical expandable prosthetic titanium rib (VEPTR) device and magnetically controlled growing rod (MCGR). However, repeated surgeries are often required, and a high complication rate between 24 and 50% has been reported [6,7,8]. Serial casting with subsequent bracing was shown to have satisfactory results in young children with curves less than 60°. It was less effective in older children, with 35% requiring spinal fusion at a mean age of 10 [9], and it can have a negative impact on childhood activities. Bracing, in contrast, is more convenient and can be removed for bathing, exercise, and other activities. Therefore, bracing is recommended as a first-line treatment for patients with idiopathic scoliosis to control the curve from progression to surgical threshold or delay surgical intervention until the adolescent years. Although the efficacy of bracing in patients with adolescent idiopathic scoliosis (AIS) is well documented, the effects of bracing for patients with EOIS to control the curve from progression to surgical threshold remains an uncharted area with little evidence available in the literature. The purpose of this longitudinal retrospective cohort study was to examine the clinical outcomes of bracing for patients with EOIS.

## 2. Materials and Methods

### 2.1. Subjects

This was a longitudinal retrospective cohort study. A series of more than 20,000 patient records during the period from 1988 to 2021 were retrieved from a scoliosis clinic in a local hospital and reviewed. In total, 111 subjects fulfilling the inclusion criteria were included in the study: (1) subjects with an onset of idiopathic scoliosis below the age of 10, diagnosed by clinical examination and a standard standing posteroanterior radiograph of the whole spine; (2) received full-time underarm thoracolumbar sacral orthosis bracing treatment until skeletal maturity (advised to wear at least 20 h a day); (3) available clinical data of curve features; (4) longitudinal follow-up every 6 months and past skeletal maturity at last follow-up, which was defined as either ≥2 years post-menarche for girls or <1 cm growth in body height in recent 6 months for both genders for intention-to-treat analysis. The general bracing practice for skeletally immature subjects indicated the following: (1) Cobb angle > 25° or (2) progression of Cobb angle > 5° in the past 6 months and with Cobb angle > 20°.

Subjects were excluded if they did not receive bracing or had a diagnosis of scoliosis with any known etiology, such as congenital scoliosis, neuromuscular scoliosis, scoliosis of metabolic etiology, scoliosis with skeletal dysplasia, known endocrine and connective tissue abnormalities, known heart condition or other diseases that could affect the safety of exercise, eating disorders or gastrointestinal malabsorption disorders and currently taking medication that affects bone or muscle metabolism.

Demographic data including gender, age, onset of menarche and skeletal maturity at diagnosis defined by Risser Sign, the duration for bracing, and history of surgery were recorded. The following clinical features, such as curve type, Cobb angle at various time points and in-brace percentage correction, were also obtained.

The Cobb angle was obtained using a standard standing posteroanterior radiograph of the whole spine using the Cobb method [10]. For subjects with more than one curve, the Cobb angle of the largest curve was used for data analysis. The Cobb angle was assessed at various time points, including the following: (i) when scoliosis was first diagnosed (initial Cobb angle); (ii) just pre-bracing; (iii) first in-bracing; (iv) just post-bracing; and (v) final follow-up (final Cobb angle). To determine whether or not the curve had progressed, the Cobb angle at the final follow-up (final Cobb angle) was dichotomized into <50° or ≥50° [11]. The Cobb angle of 50°, which often represents the lower limit of curve severity for surgery, was selected to define the success of bracing clinical outcomes.

The in-brace percentage correction was defined as follows:

(Cobb angle just pre-bracing − Cobb angle first in-bracing) ÷ (Cobb angle just pre-bracing) × 100%

All procedures performed in studies involving human participants were in accordance with the ethical standards of The Joint Chinese University of Hong Kong—New Territories East Cluster Clinical Research Ethics Committee (reference no.: CREC-2017.217) and the 1964 Helsinki Declaration and its later amendments or comparable ethical standards. Written informed consent was obtained from all subjects included in this study and their guardians before undertaking the evaluations.

### 2.2. Statistical Analysis

Pearson correlation was used to show the association between the final Cobb angle, in-brace percentage correction, and independent variables, including gender, age, curve features, bracing duration, and Cobb angle at various time points. The point-biserial correlation was used to show the association between the final Cobb angle ≥50° variables and the above-mentioned independent variables. The chi-square test was used to examine the association between curve progression and the initial Cobb angle. The Independent sample Kruskal–Wallis test was used to determine the differences in the final Cobb angles among various curve types at diagnosis. SPSS version 26.0 was used for data analysis, and *p* < 0.05 was adopted as the general level of significance.

## 3. Results

### 3.1. Demographics

Among the 20,000 records retrieved and reviewed, there were only 140 patients with an onset of idiopathic scoliosis below the age of 10, comprising only 0.7% of all scoliosis cases presented in the clinic. Of the 111 patients with EOIS who received bracing treatment, 95 were female (85.6%) and 16 were male (14.4%). The mean age at first diagnosis was 8.60 ± 1.25 years old with an average follow-up period of 9.41 ± 4.04 years up to a mean age of 18.01 ± 3.89 years old. The mean body height at diagnosis was 131.05 ± 10.88 cm. All patients were skeletally immature, with 99.1% at Risser sign stage 0 when first diagnosed. Regarding female patients with EOIS, they were on average 3.42 ± 1.96 years before menarche at diagnosis and had an average of 5.91 ± 4.06 years of menarche at the final follow-up. More patients with EOIS had the largest curve on the left side (60.4%) at first diagnosis. Most of them had a double major curve type (42.3%), followed by thoracolumbar (18.9%) and single thoracic (16.2%). The average initial Cobb angle was 21.73 ± 7.92°. Patients started bracing at a mean age of 9.74 ± 1.72 years and stopped at a mean age of 15.01 ± 1.93 years. The average bracing duration was 5.27 ± 1.93 years. Only 26 patients (23.4%) eventually underwent surgery. Most patients (60.4%) had the largest Cobb angle ranged between 15 and 29°, and only one of them had a Cobb angle that exceeded 45° at diagnosis. The demographics of patients with EOIS are summarized in Table 1.

### 3.2. Cobb Angle at Various Timepoints

The mean of the final Cobb angle was 39.78 ± 20.91°, with a mean of 18.05 ± 19.11° with respect to Cobb angle progression from diagnosis to final follow-up. An average of 31.33% ± 29.59 with respect to in-brace percentage correction was achieved, and the Cobb angle's first post-bracing was 38.42 ± 21.05°. In total, 78.1% of patients had Cobb angles that progressed more than 5°, and 66.7% of them had a final Cobb angle < 50°. The change in Cobb angle at various time points is presented in Table 2.

### 3.3. Correlations of Bracing Clinical Outcomes

The correlations between the bracing clinical outcomes of curve progression, curve features, gender and maturity are shown in Table 3. The final Cobb angle had a significant but weakly positive correlation with the curve type at diagnosis (r = 0.206), the direction of the largest curve (r = 0.342), the initial Cobb angle (r = 0.398), the first in-bracing (r = 0.328, *p* < 0.01) and the first stopped bracing (r = 0.955). It was negatively correlated with duration from menarche to diagnosis (r = −0.256), age when bracing started (r = −0.278) and body height when bracing started (r = −0.320). Similarly, the curve progression at the final follow-up with a Cobb angle of ≥50° had a weak positive correlation with the initial Cobb angle (r = 0.313), just pre-bracing, (r = 0.334), first in-bracing (r = 0.213) and first stopped bracing (r = 0.771), and it was negatively correlated with duration from menarche to diagnosis (r = −0.268), age when bracing started (r = −0.233) and body height when bracing started (r = −0.232). The in-brace percentage correction also showed a significant negative correlation with age when bracing started (r = −0.194, *p* = 0.045), body height when bracing started (r = −0.203) and direction of largest curve (−0.309) but had a positive correlation with bracing duration (r = 0.207).

**Figure 1:**
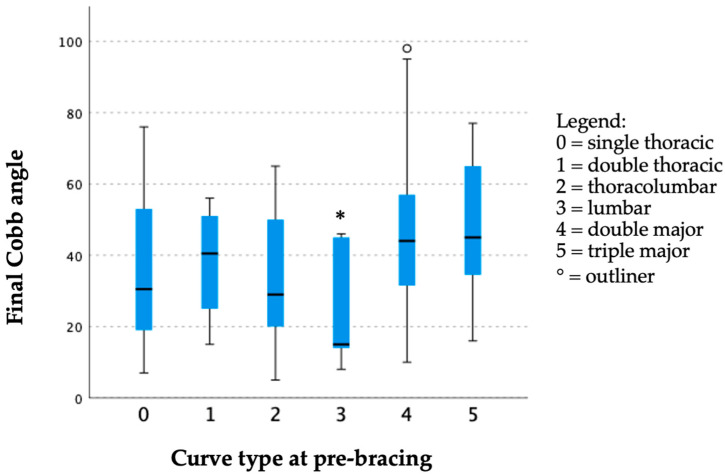
Box plot of the final Cobb angle corresponding to various curve types with just pre-bracing. Curve type at pre-bracing was shown to have a significant effect to final Cobb angle (H: 11.927, df: 5, *p* = 0.036 *) with lumbar curve type just before bracing was found to have smallest Cobb angle at last follow-up.

**Figure 2:**
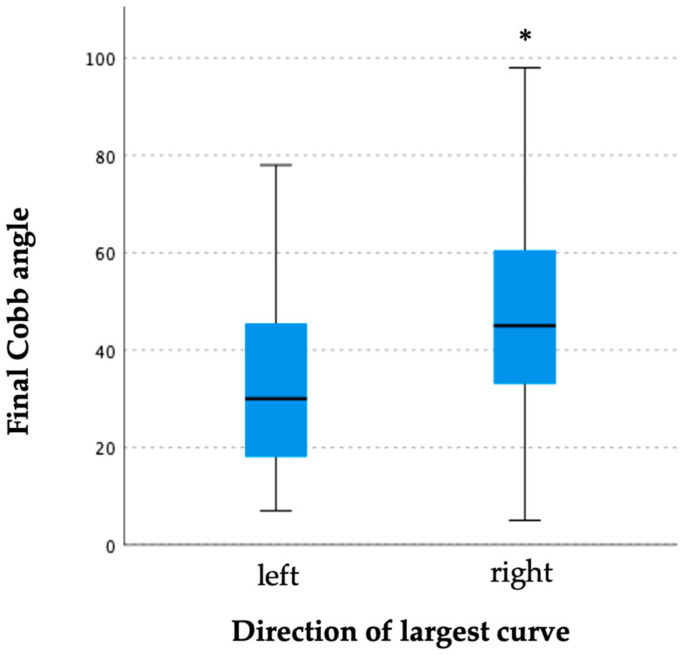
Box plot of final Cobb angle corresponding to direction of the largest curve. Direction of largest curve was shown to have a significant effect to final Cobb angle (H: 14.262, df: 1, *p* < 0.001 *) with right-sided curves observed to have a larger final Cobb angle than left-sided curves.

The curve progression at the final follow-up with a Cobb angle of ≥50° was found to be significantly correlated with the initial Cobb angle (Table 4). More curve progression was observed with higher initial curve severity at the time of diagnosis. The lumbar curve type with just pre-bracing (25.60 ± 18.37°) was found to have the smallest final Cobb angle followed by thoracolumbar (33.33 ± 17.65°) and single thoracic curve (34.44 ± 19.44°) types (Figure 1). Right-sided curves were also observed to have a larger final Cobb angle than left-sided curves (47.40 ± 19.95° vs. 33.29 ± 18.90°) (Figure 2). Patients with right-sided curves were slightly older than those with left-sided curves (8.75 ± 1.25 years vs. 8.42 ± 1.24 years).

## 4. Discussion

The management of EOIS remains challenging as the selection of the best management strategy is often complicated by the heterogeneity of this population, and no known treatment option is superior [5]. The average age at diagnosis of our subjects is 8.6 years old, which coincided with the consensus of expert surgeons in identifying “tweeners”, a terminology coined by Quan et al., referring to a subset of patients with early-onset scoliosis [12]. The definition of “tweeners” is an important milestone for differentiating this rare group of subjects from a more prevalent and well-studied population of idiopathic scoliosis adolescents. Bracing plays an important role in the non-operative treatment for managing idiopathic scoliosis. The current study is an important contribution to bracing research as very few studies evaluated the clinical outcomes of bracing for idiopathic scoliosis with early onset.

In total, 66.7% of the patients in this cohort had a final Cobb angle of <50°, which was below the surgical threshold and defined by previous studies as a successful bracing outcome [11,13,14]. Furthermore, for patients with an initial Cobb angle of <45°, only less than 25% of them would progress beyond 50°. However, the mean of the final Cobb angle was nearly 40°, with a mean Cobb angle of 18.05° progressing from diagnosis to final follow-up, which suggested that a considerable portion of patients in this cohort would progress to a moderate curve severity but fortunately not to the extent of reaching a surgical threshold. An increase in the initial Cobb angle was observed with a higher final Cobb angle and with a curve progression of >50°, which was similar to the findings by Sitoula et al. [11] and Karol et al. [15] in their studies of mixed-gender juvenile, preadolescent and adolescent idiopathic scoliosis populations. The Cobb angle after the first post-bracing in this cohort was 38.42°, which is below the important cut-off of 40° for curve progression beyond skeletal maturity [13,16].

Our results also showed that the final Cobb angle was negatively correlated with menarche, chronological age and body height when bracing started. These maturity indicators have been reported to be closely correlated with skeletal growth and curve progression [17]. The longer duration from menarche to diagnosis, younger age and shorter body height when bracing started was shown to have a larger final Cobb angle and greater curve progression exceeding 50°.

Several studies suggested that at least a 35–50% in-brace correction is required to prevent significant curve progression [18,19,20]. These studies were conducted mostly in populations of patients with AIS, in which factors such as spinal flexibility, the amount of vertebral rotation and skeletal maturity could affect the achievable in-brace correction. The average in-brace percentage correction was only 31.33% in the current study, which did not reach the standards of some of these studies. Comparably, Yen et al. studied the immediate effects of in-brace correction in patients with AIS and found that only 24% of their patients achieved more than 30% in-brace correction [21]. Previous studies found the predictive value of in-brace correction for curve progression [22,23]; however, there was no correlation between in-brace correction, curve progression and the final Cobb angle in the current study. Karol et al. also found that in-brace correction could not predict curve progression in male patients with AIS [15].

The curve type was also found to be associated with the final Cobb angle. Lumbar curve types, as compared to thoracic curve types, resulted in a smaller final Cobb angle. Lenke et al. also found that main thoracic curves could predict curve progression to 50° and a higher rate of surgery after controlling compliance [24]. This result supported the findings by Dolan et al. [25] that the Cobb angle cut-offs for high curve progression risk differed between thoracic and lumbar curves. Lara et al. [26] also found that the incidence of surgery could also be predicted by curve type. However, Katz and Durrani reported that there was no difference between curve types and the risk of curve progression [22]. Patients with right-sided curves were older and noted to have a larger final Cobb angle. Since patients less than 10 years of age were recruited in this study, some of them might have entered the pre-adolescent period with rapid skeletal growth at diagnosis as the right-sided curve is the most typical presentation of AIS.

The retrospective nature of the study limited the comparison with a control group. Only a few subjects in this cohort did not receive bracing treatment due to various reasons, such as refusal of bracing. Due to the low prevalence and heterogeneity of EOIS, the small number of subjects without bracing treatment could not provide sufficient statistical power as controls for comparison, and only a single-arm study with a small sample size over a study period spreading over three decades could be conducted. Since the records were retrieved over a period of more than three decades, some data on anthropometric measurement, factors related to skeletal maturity and radiographic features at various time points were unavailable. The rarity of the samples and the lack of some data from older records limit sub-group and stratification analyses. The current study could be a start for evaluating the clinical outcomes of bracing treatment for EOIS, and further studies can be conducted to evaluate whether the side of the curve corresponds to the location of the major curve and hence the possible difference relative to in-brace correction, response to brace treatment and the final Cobb angle among various sub-types of scoliosis. The compliance of brace wearing was not rigorously monitored previously, which made further analyses for comparing bracing effects between compliant and non-compliant patients impossible. With advances in brace compliance measurements in recent years, temperature sensors can be attached to the brace and accurately record brace wearing hours and patterns. EOIS remains a rare spinal deformity, but our study could offer an average of more than 9 years of follow-up, with all patients reaching skeletal maturity.

## 5. Conclusions

Our current study has offered new insights into the clinical outcomes of bracing for EOIS. Bracing could prevent curve progression to the surgical threshold in most patients with EOIS, and the initial Cobb angle was positively correlated with the final Cobb angle. Further prospective research could include multi-centre studies with sizeable samples and stratification, radiographic features, etiopathogenetic-relevant parameters, robust monitoring of brace compliance and a longer follow-up period after weaning to monitor progression and identify determinants governing bracing effectiveness in EOIS. While bracing can help to control spinal deformity along the course of EOIS, the question of when bracing should be started remains. To answer this, it is important to identify clinical parameters related to etiopathogenetic pathways for enhancing prediction, response to bracing and other non-operative treatments.

## Figures and Tables

**Table 1:** Demographics of patients with EOIS.

		EOIS (*n* = 111)
**Gender**	Female	95 (85.6%)
	Male	16 (14.4%)
**Age**		
Age at diagnosis (years)		8.60 ± 1.25
Age at final follow-up (years)		18.01 ± 3.89
Follow-up duration (years)		9.41 ± 4.04
**Body height**		
Body height at diagnosis (cm) (*n* = 90)		131.05 ± 10.88
Body height at final follow-up (cm) (*n* = 107)		159.92 ± 13.26
**Maturity**		
Risser sign (*n* = 108)	Stage 0	107 (99.1%)
	Stage 1	1 (0.9%)
Duration from menarche to diagnosis (years) (*n* = 93)		−3.42 ± 1.96
Duration from menarche to final follow-up (years) (*n* = 93)		5.91 ± 4.06
**Treatment received**		
Age when started bracing (years)		9.74 ± 1.72
Age when stopped bracing (years)		15.01 ± 1.56
Bracing duration (years)		5.27 ± 1.93
Underwent surgery		26 (23.4%)
**Curve Features**		
Side of largest curve at diagnosis	Left	67 (60.4%)
	Right	44 (39.6%)
Curve type just pre-bracing	Single thoracic	18 (16.2%)
	Double thoracic	4 (3.6%)
	Thoracolumbar	21 (18.9%)
	Lumbar	5 (4.5%)
	Double major	47 (42.3%)
	Triple major	16 (14.4%)
Initial Cobb angle (°)		21.73 ± 7.92
Largest initial Cobb angle (°)	10–14°	23 (20.7%)
	15–29°	67 (60.4%)
	30–44°	20 (18%)
	≥45°	1 (0.9%)

The values are presented as number and percentage (%) and mean ± standard deviation as appropriate.

**Table 2:** Number, percentage and mean of Cobb angle at various time points.

		EOIS (*n* = 111)
Cobb angle just pre-brace (°)		24.74 ± 6.65
Cobb angle 1st in-brace (°)		16.94 ± 8.62
Cobb angle 1st post-brace (°)		38.42 ± 21.05
Final Cobb angle (°)		39.78 ± 20.91
Cobb angle progression from diagnosis to final follow-up (°)		18.05 ± 19.11
Cobb progression ≤5° at final follow-up (*n* = 110)		31 (28.2%)
Cobb progression >5° at final follow-up (*n* = 110)		79 (71.8%)
In-brace percentage correction (%)		31.33 ± 29.59
Largest final Cobb angle (°)	10–14°	10 (9%)
	15–29°	24 (21.6%)
	30–44°	29 (26.1%)
	45–49°	11 (10%)
	≥50°	37 (33.3%)

The values are presented as number and percentage (%) and mean ± standard deviation as appropriate.

**Table 3:** Correlation between bracing clinical outcomes of curve progression, curve features, gender and maturity.

EOIS (*n* = 111)	Final Cobb Angle ^a^		In-Brace Percentage Correction ^a^		Final Cobb Angle ≥ 50 ^b^	
	r	*p*-Value	r	*p*-Value	r_bp_	*p*-Value
Gender	−0.038	0689	0.064	0.513	0.036	0.705
Age at diagnosis	−0.041	0.672	−0.070	0.472	−0.083	0.385
Duration from menarche to diagnosis	−0.0256	0.013 *	−0.113	0.289	−0.268	0.009 **
Curve type at diagnosis	0.206	0.030 *	−0.021	0.833	0.048	0.621
Curve type just pre-bracing	0.268	0.004 **	−0.097	0.323	0.118	0.217
Age when bracing started	−0.278	0.003 **	−0.194	0.045 *	−0.233	0.014 *
Bracing duration	0.178	0.061	0.207	0.032 *	0.155	0.105
Body height when bracing started	−0.320	0.001 **	−0.203	0.040 *	−0.232	0.017 *
Direction of largest curve	0.342	0.000 **	−0.309	0.001 **	0.192	0.044
Initial Cobb angle	0.398	0.000 **	0.081	0.405	0.313	0.001 **
Cobb angle just pre-bracing	0.388	0.000 **	0.102	0.295	0.334	0.000 **
Cobb angle first in-bracing	0.328	0.001 **	−0.744	0.000 **	0.213	0.028 *
Cobb angle first stopped bracing	0.955	0.000 **	−0.170	0.080	0.771	0.000 **
In-brace percentage correction	−0.116	0.232			−0.030	0.763

** *p* ≤ 0.01; * *p* ≤ 0.05; ^a^: Pearson correlation (r) was used on continuous data. ^b^: Point-biserial correlation (r_bp_) was used on categorical data.

**Table 4:** Correlation between the Cobb angle at first diagnosis and curve progression.

EOIS (*n* = 111)	Final Cobb Angle < 50°	Final Cobb Angle ≥ 50°	*p*-Value
Initial Cobb angle	*n* (%)	*n* (%)	<0.001 **
10–14°	3 (75%)	1 (25%)	
15–29°	16 (78.9%)	3 (21.1%)	
30–44°	57 (74.6%)	10 (25.4%)	
≥45°	9 (28.6%)	12 (71.4%)	

** *p* ≤ 0.01. The values are presented as numbers and percentages (%).

## Data Availability

The data presented in this study are available on request from the corresponding author. The data are not publicly available due to patient privacy and confidentiality.
